# Durability study of Veeralin^®^, Olyset^®^ Plus and MAGNet^®^ insecticide-treated nets (ITNs) in Kurnool district, Andhra Pradesh, India

**DOI:** 10.1186/s13071-025-07016-2

**Published:** 2025-10-29

**Authors:** Vani Hanumantappa Chalageri, Shrinivasa B. Marinaik, N SujithNath, Sreehari Uragayala, Vaishali Verma, S. P. Singh, Rajendra Baharia, Diamond P. Sinha, G. Shankar, Richa Singhal, Alex Eapen, Emmanuel Mbuba, Sarah J. Moore, Raghavendra Kamaraju, Anupkumar Anvikar

**Affiliations:** 1 ICMR-National Institute of Malaria Research, Sector 8, Dwarka, New Delhi 110077 India; 2 Vector Control Product Testing Unit (VCPTU), Environmental Health and Ecological Sciences, Ifakara Health Institute, P.O. Box 74, Bagamoyo, Tanzania; 3 Swiss Tropical and Public Health Institute, Kreuzstrasse 2, 4123 Allschwil, Switzerland; 4 University of Basel, Petersplatz 1, 4001 Basel, Switzerland; 5 The Nelson Mandela African Institute of Science and Technology (NM-AIST), P.O. Box 447, Tengeru, Tanzania; 6 Faculty of Biological Sciences, AcSIR, Ghaziabad, Uttar Pradesh India; 7 H. No. 104, BSF Signal Training School, Yelahanka, Bangalore, Karnataka 560064 India; 8 Present Address: H. No. 28B, Block ED, Pitampura, Delhi India

**Keywords:** Veeralin^®^ ITNs, MAGNet^®^ ITNs, Olyset^®^ Plus ITNs, Insecticide-treated nets (ITNs), Piperonyl butoxide, Durability

## Abstract

**Background:**

India plans to eliminate malaria by 2030, targeting zero local cases by 2027. However, malaria remains prevalent in endemic regions. As a means of addressing this issue, insecticide-treated nets (ITNs) are crucial for prevention. In areas with pyrethroid-resistant vectors, studies indicate that ITNs containing pyrethroids and piperonyl butoxide (PBO) are more effective. However, their physical and chemical durability and bioefficacy in this setting require further evaluation. This study assessed the durability of two pyrethroid–PBO-treated nets and one pyrethroid-only-treated net to determine whether they met the World Health Organization (WHO)’s 3-year operational performance criteria for “long-lasting” classification.

**Methods:**

A total of 7570 ITNs—2664 Veeralin^®^ (alpha-cypermethrin and PBO), 2246 Olyset^®^ Plus (permethrin and PBO), and 2660 MAGNet^®^ (alpha-cypermethrin)—were distributed among 4186 households, following village randomization in Kurnool district, Andhra Pradesh, India. Following the baseline survey, all distributed ITNs were followed up annually for 36 months to measure ITN survival and bioefficacy.

**Results:**

The median functional survival period of the ITNs was estimated at 8 years (interquartile range [IQR] 7.00–9.00 years) for Veeralin^®^, 5.67 (IQR 5.00–6.33) years for MAGNet^®^, and 2.51 (IQR 2.19–2.88) years for Olyset^®^ Plus. Bioassays conducted 36 months post-distribution using susceptible mosquito strains revealed 24-h mortality rates of 93.5% (91.8–95.3) for Veeralin^®^ ITNs, 45.0% (33.7–56.4) for MAGNet^®^ ITNs, and 59.3% (50.7–68.70) for Olyset^®^ Plus ITNs. The “optimally insecticidal” period (24-h mortality > 80%) was 30 months for Veeralin^®^ ITNs (84.0% [77.3–90.9] mortality), 18 months for MAGNet^®^ ITNs (94.6% [92.2–97.2] mortality), and 12 months for Olyset^®^ Plus ITNs (98.2% [97.5–98.9] mortality). Bioassays using an alpha-cypermethrin-resistant strain at 36 months showed 68.5% (64.9–72.0) mortality for Veeralin^®^ ITNs and 58.1% (52.0–64.2) mortality for Olyset^®^ Plus ITNs. Community acceptability at 36 months was 88.5% for Veeralin^®^ ITNs, 86.7% for MAGNet^®^ ITNs, and 56.3% for Olyset^®^ Plus ITNs.

**Conclusions:**

In the Indian geographic setting, Veeralin^®^ ITNs met the criterion to be considered long-lasting insecticidal nets, remaining in serviceable condition (96.1%) for more than 3 years. They remained optimally insecticidal against pyrethroid-resistant mosquitoes and susceptible mosquitoes for 3 years.

**Graphical Abstract:**

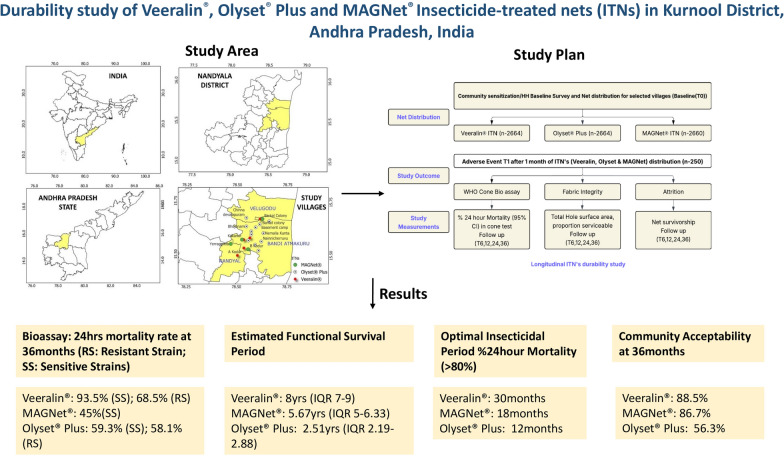

**Supplementary Information:**

The online version contains supplementary material available at 10.1186/s13071-025-07016-2.

## Background

India has made significant progress in reducing its malaria incidence since 2015 [1]. This period coincides with the launch of the National Framework for Malaria Elimination (NFME) by the Government of India (GOI) in 2016 [2]. Between 2017 and 2024, malaria cases decreased by 69% (from 6.4 million to 2 million), and deaths declined by 68% (from 11,100 to 3500) [3]. The drastic decline in malaria cases led the nation to exit the list of high-burden countries in 2024 [4]. This reduction can be largely attributed to successful interventions, including improved diagnosis, treatment, and vector-control interventions [1]. Nonetheless, certain endemic regions have shown sustained malaria incidence since 2020. In 2022, India still accounted for 66% of the malaria burden in Southeast Asia. Malaria continues to be a significant concern for public health, causing considerable morbidity and mortality, particularly in rural and remote areas. Insecticide-treated nets (ITNs) constitute a crucial component of malaria prevention strategies, effectively providing a barrier against mosquito bites, killing them upon contact, and consequently reducing the incidence of malaria [5].

The widespread use of pyrethroid insecticides has contributed to the development of resistance in mosquito populations, thus affecting the efficacy of ITNs [6, 7]. Piperonyl butoxide (PBO), a synergist, enhances the effectiveness of pyrethroids by inhibiting the metabolic enzymes responsible for the detoxification of pyrethroids in metabolically resistant mosquitoes, thereby increasing the insecticidal potency of interventions against resistant mosquito strains [8]. PBO nets may be useful for controlling mosquitoes with metabolic resistance to pyrethroids [9, 10], and incorporating PBO into ITNs has been shown to enhance malaria control in Africa [8]. This study focused on the significance of PBO in controlling both pyrethroid-sensitive *Anopheles stephensi* and pyrethroid-resistant *Anopheles culicifacies*—two of the primary malaria vectors in India [11]—and evaluated its potential to intensify control efforts in India against emerging pyrethroid resistance in malaria vectors [12].

In India, where the distribution of ITNs has been extensive, understanding their durability is essential for maintaining the effectiveness of malaria control programs. The physical condition and insecticidal potency of ITNs decline over time, potentially influencing malaria transmission [13, 14]. Therefore, assessing the durability of ITNs in real time is crucial for guiding policy decisions, optimizing resource allocation, and improving the design and distribution strategies of ITN programs [15]. Durability is vital for ensuring both long-term effectiveness and cost-efficiency [16]. It includes both the physical integrity and persistence of the insecticidal properties of the nets [17, 18]. Various factors influence the durability of ITNs, such as the quality of the netting material [19], washing frequency [20], exposure to sunlight [21], and general wear and tear from routine use [22]. This study aimed to evaluate the durability of three ITNs in India by examining their physical integrity and insecticidal activity over 3 years. The three nets assessed were Veeralin^®^ and Olyset^®^ Plus ITNs (PBO-treated), which were compared against MAGNet^®^ ITNs (pyrethroid-only, used as a control). This research provides valuable insights for the optimization of malaria prevention strategies aimed at achieving malaria elimination in India.

## Methods

This longitudinal study followed nets distributed at the village level (covering all households per village) for over 3 years [23]. The project was initially planned for 3 years but took longer to complete due to the COVID-19 pandemic and lockdown restrictions, which impacted field evaluations and follow-ups. The procedures used to assess ITN durability were conducted following World Health Organization (WHO) guidelines [24, 25]. The median functional survival and ITN efficacy were evaluated with the endpoints listed in Supplementary Table 1. The attrition, net damage, and insecticidal efficacy components were tested at various time points throughout the study period. The study was approved by the Institutional Scientific Advisory Committee of NIMR [National Institute of Malaria Research (ECR/NIMR/EC/2018/99)] and the Health Minister’s Screening Committee of India. Written informed consent was obtained from the participants in Telugu (the local language) before the baseline survey was conducted.

### Insecticide-treated nets

The Veeralin^®^ ITNs are 130-denier monofilament polyethylene ITNs, manufactured by V.K.A. Polymers Pvt. Ltd., India. These ITNs are incorporated with alpha-cypermethrin at a target dose of 6.0 g AI/kg (equivalent to 216 mg/m^2^), and with PBO at 2.2 g/kg (equivalent to 79.2 mg/m^2^). The ITNs are listed as pre-qualified (PQ).

The Olyset^®^ Plus ITNs are 150-denier knitted monofilament polyethylene ITNs, manufactured by Sumitomo Chemical, Japan. These PQ-listed ITNs are incorporated with permethrin at a target dose of 20 g/kg (equivalent to 800 mg/m^2^) and PBO at 10 g/kg (equivalent to 400 mg/m^2^).

The MAGNet^®^ ITNs are 150-denier knitted monofilament polyethylene nets, manufactured by V.K.A. Polymers Pvt. Ltd., India. These nets are treated with alpha-cypermethrin at a target dose of 5.8 g/kg. They are PQ-listed.

### Study area

**Fig. 1:**
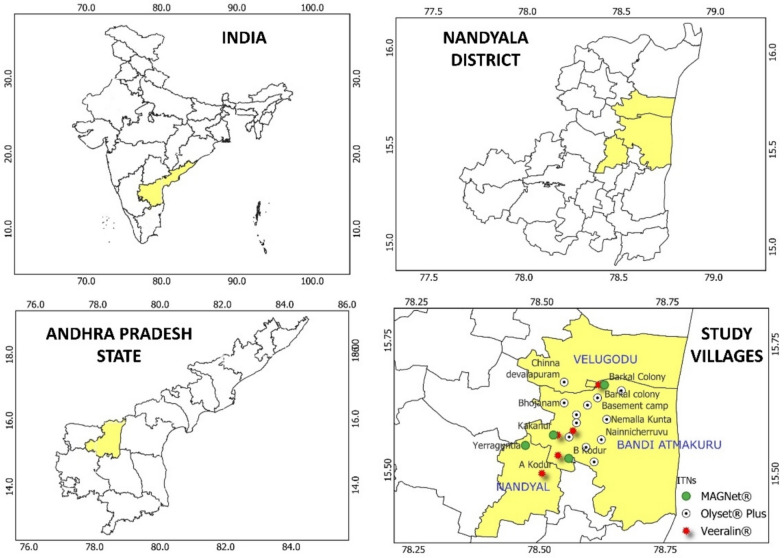
Map showing the study area (Nandyal Mandal) in the Kurnool district, Andhra Pradesh, India

The study was carried out in the Nandyal District of Andhra Pradesh, India (Fig. 1). It lies between 15.47°N latitude and 78.48°E longitude. The villages involved in the study are situated along the embankments of the River Tungabhadra. The region has a tropical climate, with summer temperatures varying between 26 °C (78.8 °F) and 46 °C (114.8 °F), and winter temperatures ranging from 12 °C (53.6 °F) to 31 °C (87.8 °F). The average yearly rainfall in the area is approximately 705 mm. The density of *An. culicifacies* mosquitoes is very high at the study sites due to the river’s proximity, leading the community to use ITNs regularly to prevent mosquito bites.

### Study implementation

In September 2019, households were enumerated, and the number of nets to be distributed to each household was calculated on the basis of the formula provided by the National Center for Vector Borne Diseases Control (NCVBDC), Ministry of Health & Family Welfare, GOI. ITNs were distributed considering the sleeping patterns of inhabitants. Couples received a single net, which also covered two children under the age of 10. Each pregnant woman and elderly individuals were provided with a separate net [26]. At the beginning of the study, community sensitization programs were conducted to inform participants about the study's purpose and to guide them in the proper care and use of ITNs. A baseline survey was conducted across all the selected villages via a structured questionnaire on the Open Data Kit (ODK) platform to collect household-level information. Additionally, an adverse event survey was conducted 1 month after the baseline survey.

Each net was labeled with a unique identifier (UID). A master list of the household IDs was prepared at baseline, featuring six numbers generated by the study statistician: the first number identified the country, the second identified the treatment arm, and the remaining four numbers identified the nets distributed per arm. Two lists were generated via a random number generator from the household master list to assess attrition and fabric integrity, and another list was generated for destructive sampling for bioefficacy and chemical retention monitoring.

From the households enumerated in the baseline survey (with each ITN assigned a unique net ID), a subsample of 30 nets (of each type of ITN) was randomly selected and withdrawn for bioefficacy testing via the WHO cone test procedure every 6 months for 30 months, and again at the 36-month time point, where an additional 50 nets were withdrawn. If a randomly selected ITN was unavailable in the household, the next listed ITN for that particular household was chosen. If no study ITNs were available in the household, another nearby household with the same type of net was visited, and the same selection process was followed. ITNs withdrawn for destructive sampling were replaced with new ITNs of the same type. The households from which ITNs were collected for analysis were removed from the master list of enumerated households to prevent newly distributed ITNs from being selected for analysis at subsequent time points.

### Sample size

The sample size was calculated on the basis of the number of households to be followed up for attrition at 6 months and then annually for up to 3 years. The target sample size was 328 households per arm. Assuming an average of 2.7 nets per household and a coefficient of variation of 0.25 (to account for clustering by household), the formula on page 110 of Hayes and Moulton [27] indicated that this target sample size would allow detection of a difference in attrition at 36 months of 52.5–44.1%, with 80% power at the 5% significance level. The target sample size was larger than the WHO 2013 guidelines [28], which require at least 250 nets per arm for each time point. A maximum of three nets were randomly selected in each household and inspected for attrition and holes. For bioassays at each time point, an average of 30 ITNs (one net per household) were tested for the sensitive strain. However, due to the low number of resistant-strain mosquitoes at some time points, fewer samples were tested.

### Attrition assessment

Attrition monitoring was performed at 6, 12, 24, and 36 months, during which the study ITNs in each visited household were inspected for attrition and fabric integrity. All study ITNs listed in the master list generated at baseline were checked for their presence, and those present in the household were inspected for damage [29]. The presence or absence of all distributed ITNs was noted, and if any were missing, the head of the household was asked to provide reasons for their absence. ITNs that were lost due to wear and tear were classified as “attrite,” whereas ITNs that were missing because they were stolen, given away to others, or sold were treated as “lost to follow-up.”

### Monitoring fabric integrity

Each ITN found during attrition monitoring was assessed for damage by counting the number of holes of four different sizes, namely 0.5–2 cm, 2–10 cm, 10–25 cm, and above 25 cm, approximated using a thumb, fist, head, and larger than a head, respectively [28]. The total physical condition of the ITNs was obtained by estimating the total hole surface area (THSA) and weighting the number of holes of each size by the factors 1.23, 28.28, 240.56, and 706.95, respectively, and then summing them following WHO guidelines. The conditions of the ITNs were categorized as “good” (< 79 cm^2^ THSA), “damaged” (79–788 cm^2^ THSA), or “severely torn” (≥ 789 cm^2^ THSA). According to the WHO recommendation, an ITN was considered “serviceable” if it was classified as good or damaged [25, 30].

### Bioefficacy assessment

Each ITN collected from the field sites was initially cross-checked at the Indian Council of Medical Research (ICMR)-NIMR, Field Unit-Bangalore. From these, eight small pieces (each measuring 25 cm × 25 cm) were cut for conducting cone bioassays and chemical analysis (four for bioassays and four for chemical analysis). The sample cutting and storage process was performed according to WHO guidelines [28].

### Mosquito colony maintenance

Three strains of pyrethroid-susceptible *An. stephensi* were used: Pondicherry, Sonipat, and Devanahalli. The pyrethroid-resistant strains used in the study were first filial generation (F_1_) and field-collected *An. culicifacies*. All the colonies were maintained at 29–30 °C and 75–80% relative humidity, with a 10 h:14 h light/dark cycle. Eggs were bleached with 0.1% formalin and 1% NaOH to prevent infection. Larvae were kept at a density of approximately 250 per tray in 1.5 L of reverse osmosis water for early instars and 2 L for later instars. Finely ground fish food (TetraBits) was prepared, labeled, and stored at 4 °C in a refrigerator once a week to avoid infection and contamination. Inactive, infected, and dead larvae were removed to prevent infection. Adults were kept in cages measuring 30 cm × 30 cm × 30 cm, with approximately 250 mosquitoes per cage. Adult mosquitoes were fed soaked raisins and a 10% glucose solution made with sterile water ad libitum. Blood meals were provided by allowing mosquitoes to feed on restrained mice and later via an artificial membrane. The mosquito resistance profile is provided in Supplementary Table 2.

### Cone assays

To perform the cone bioassays, standard WHO protocols were followed [25], with the angle of the board set at 45°, and no hole cut out from the testing board. Over each net sample, a standard WHO cone, procured from the WHO, was placed and held using masking tape. The cones were stopped with a latex bung to prevent the mosquitoes from resting on the stopper. Five laboratory-bred, sugar-fed, 2–5-day-old susceptible mosquitoes were placed into each cone and exposed for 3 min. A control with untreated nets was run for each strain during each experiment to assess the quality of the bioassay. Bioassays were conducted daily between 11 AM and 4 PM to avoid bias from known changes in mosquito metabolism throughout the day.

After exposure, the mosquitoes were transferred to 750-ml plastic holding cups (one cup per cone test replicate) and provided with a 10% sugar solution moistened with cotton wool. Plastic holding cups were covered with untreated netting, with a small hole cut into the top to allow for aspiration and removal of mosquitoes. The hole was stopped with cotton wool. Fresh paper cups were used for each test. Knockdown (KD) was recorded at 60 min (KD60), and mortality was recorded at 24 h (M24) post-exposure. The bioassays and holding procedures were conducted at a median temperature of 27 °C ± 2 and a median of 75–80% relative humidity. The 24-h mortality rates in the control replicates were generally within the acceptable range; where needed, Abbot’s corrections [25] (control mortality > 20% was rejected) were applied.

### Data analysis

Demographic characteristics were summarized using proportions for categorical variables, and medians and interquartile ranges (IQRs) or means and standard deviations (SDs) were used for continuous variables. Binary outcomes (attrition, functional survival, KD, and mortality) were summarized with counts and percentages for each time point and arm. The mean control-corrected mortality was summarized as the mean value per net and presented with 95% confidence intervals (CIs) calculated via the normal approximation. The THSA was summarized as the median and IQR and compared via the Mann‒Whitney *U*-test. The serviceability of different net types over different time points (12, 24, and 36 months) was analyzed using odds ratios (ORs) and CIs. Functional survivability was calculated as the difference in the proportion of serviceable nets (analyzed with ORs and CIs) [11]. The median functional survival was calculated via the GIGA calculator [31].

## Results

**Fig. 2:**
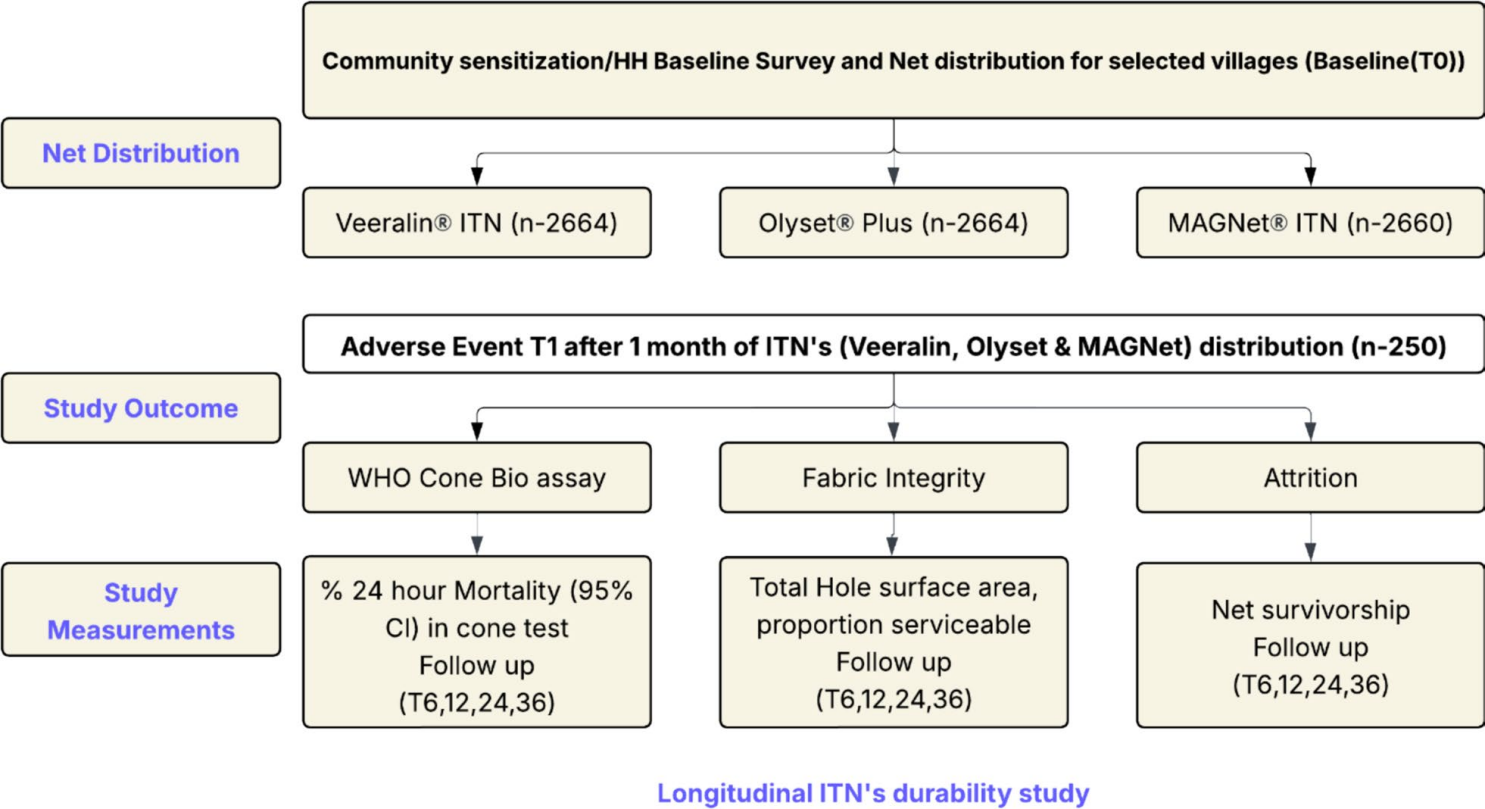
Flowchart of the study plan implemented

A total of 7570 ITNs were distributed to 4186 households across different villages, following village-level randomization, in the Kurnool district of Andhra Pradesh. The distributed nets included 2664 Veeralin^®^ ITNs, 2660 MAGNet^®^ ITNs, and 2246 Olyset^®^ Plus ITNs (Fig. 2). The villages were selected such that the demographics of the study arms were similar (Supplementary Table 3).

### Attrition and functional survival

At 36 months post-distribution, the functional survival rates were 85.5% for Veeralin^®^ ITNs, 38.6% for Olyset^®^ Plus ITNs, and 69.6% for MAGNet^®^ ITNs (Table 1). The analysis revealed that the estimated median functional survival period was greater than 36 months for Veeralin^®^ ITNs, i.e., 8 years (95% CI, IQR 7.00–9.00), and MAGNet^®^ ITNs, i.e., 5.67 years (95% CI, IQR 5.00–6.33). In contrast, the median survival for Olyset^®^ Plus ITNs was 2.51 years (95% CI, IQR 2.19–2.88). Veeralin^®^ ITNs demonstrated significantly greater functional survival than Olyset^®^ Plus ITNs (OR: 9.46; 95% CI 6.32–14.19; *P* < 0.001).

**Table 1:** Functional survival of MAGNet^®^ ITNs, Veeralin^®^ ITNs and Olyset^®^ Plus ITNs

	MAGNet^®^ ITNs				Veeralin^®^ ITNs				Olyset^®^ Plus ITNs				Odds ratio Veeralin^®^ ITNs to Olyset^®^ Plus ITNs		
Post-distribution time point (months)	Number distributed ^a^	Number in household	Number serviceable	Functional survival (%)	Number distributed ^a^	Number in household	Number serviceable	Functional survival (%)	Number distributed ^a^	Number in household	Number serviceable	Functional survival (%)	OR for functional survival^b^	95% CI	*P*-value
12	585 (575)	548	542	94.3	541 (530)	492	483	91.13	531 (526)	459	451	85.74	1.69	1.12–2.55	0.005
24	528 (524)	396	376	71.8	576 (574)	478	469	81.71	521 (510)	433	406	79.61	1.14	0.82–1.60	0.215
36	570 (566)	496	394	69.6	555 (552)	491	472	85.51	435 (430)	193	166	38.6	9.46	6.32–14.19	< 0.001

^a^Number is based on the houses visited at each time point, which varied slightly at each survey (excludes those given away/sold/stolen)

^b^Odds ratio is presented with the probability of Veeralin^®^ ITNs found in a serviceable condition compared to Olyset^®^ Plus ITNs as the reference with 95% CI

The community acceptability (after 36 months) for Veeralin^®^ ITNs was 88.5% (491/555 households), which was similar to that of MAGNet^®^ ITNs, at 86.7% (494/570 households). However, the Olyset^®^ Plus ITNs had lower acceptability of 56.9% (245/430 households).

### Physical damage

All nets available in households (Table 1) during follow-up surveys at various time points in the study were assessed, and only those with at least one hole were assessed for damage. A substantial difference in the THSA among the three ITN types was observed at each time point (Table 2). Differences in functional survival were likely due to differences in damage. At 24 months, the THSA of MAGNet^®^ ITNs was significantly lower than that of Olyset^®^ Plus ITNs (*P* < 0.001). Significant differences in the THSA were also observed between Veeralin^®^ and Olyset^®^ Plus ITNs at both 24 months (*P* = 0.003) and 36 months (*P* < 0.001) (Table 2), with Veeralin^®^ resulting in less damage.

**Table 2:** Physical integrity of ITNs: total hole surface area (median and interquartile range) of the field-aged nets

Total hole surface area (median with IQR) (*n* = number of nets with at least one hole)					
Time point (T) in months	MAGNet^®^ ITNs	Veeralin^®^ ITNs	Olyset^®^ Plus ITNs	*P*-value (MAGNet^®^ ITNs vs. Olyset^®^ Plus ITNs)	*P*-value (Veeralin^®^ ITNs vs. Olyset^®^ Plus ITNs)
T12	723 (241–3595) (*n* = 14)	1321 (175–2524) (*n* = 15)	560 (134–1975) (*n* = 22)	0.451	0.572
T24	159 (9.23–962.24) (*n* = 74)	205 (57–831) (*n* = 32)	883 (226–1431) (*n* = 52)	< 0.001*	0.003*
T36	707 (79–1777) (*n* = 221)	64 (28–754) (*n* = 81)	789 (128–1433) (*n* = 54)	0.957	< 0.001*

“Damaged” was defined as having at least one hole. Median and IQR surface area with holes in cm^2^

*N*: number of damaged nets assessed

* Indicates *P*-value is significant at 5% level of significance

At 36 months, a greater proportion of MAGNet^®^ ITNs had degraded (79.4% serviceable) compared to the Olyset^®^ Plus ITNs (86.0%). However, the OR of 0.628 (95% CI 0.396–0.996) indicates that Olyset^®^ Plus ITNs performed significantly better.

Among the nets remaining, most were serviceable, and there were differences in the proportion of serviceable nets between brands (Table 3). No significant difference in serviceability was observed between Veeralin^®^ and Olyset^®^ Plus ITNs at 12 months (*P* = 0.952). However, Veeralin^®^ ITNs were significantly more serviceable than Olyset^®^ Plus ITNs at both 24 months (98.1% vs. 93.8%; OR 3.465, 95% CI 1.611–7.454) and 36 months (96.1% vs. 86.0%; OR 4.041, 95% CI 2.188–7.458) (Table 3).

**Table 3:** Serviceability of different ITNs at different time points

Time point (T) in months	Net make	Serviceable	Not serviceable	Odds ratio (95% CI)
T6	MAGNet^®^ ITNs	325 (98.8%)	4 (1.2%)	2.166 (0.718 to 6.534)
	Olyset^®^ Plus ITNs	600 (97.4%)	16 (2.6%)	
T12	MAGNet^®^ ITNs	542 (98.9%)	6 (1.1%)	1.602 (0.551 to 4.652)
	Olyset^®^ Plus ITNs	451 (98.3%)	8 (1.7%)	
T24	MAGNet^®^ ITNs	376 (94.0%)	20 (6.1%)	1.250 (0.689 to 2.266)
	Olyset^®^ Plus ITNs	406 (93.8%)	27 (6.2%)	
T36	MAGNet^®^ ITNs	394 (79.4%)	102 (20.6%)	0.628 (0.396 to 0.996)
	Olyset^®^ Plus ITNs	166 (86.0%)	27 (14.0%)	
T6	Veeralin^®^ ITNs	534 (98.9%)	6 (1.1%)	2.373 (0.922 to 6.109)
	Olyset^®^ Plus ITNs	600 (97.4%)	16 (2.6%)	
T12	Veeralin^®^ ITNs	483 (98.2%)	9 (1.8%)	0.952 (0.364 to 2.488)
	Olyset^®^ Plus ITNs	451 (98.3%)	8 (1.7%)	
T24	Veeralin^®^ ITNs	469 (98.1%)	9 (1.9%)	3.465 (1.6110 to 7.454)
	Olyset^®^ Plus ITNs	406 (93.8%)	27 (6.2%)	
T36	Veeralin^®^ ITNs	472 (96.1%)	19 (3.9%)	4.041 (2.188 to 7.458)
	Olyset^®^ Plus ITNs	166 (86.0%)	27 (14.0%)	

### Bioefficacy

The quality check conducted at the baseline stage (month 0) through cone bioassays confirmed that all the ITNs met the minimum bioefficacy standards before distribution (Tables 3 and 4).

**Table 4:** Cone bioassays of ITNs at different time points using susceptible strain *Anopheles stephensi*

Survey (month)	MAGNet^®^ ITNs				Veeralin^®^ ITNs				Olyset^®^ Plus ITNs			
	%KD	%24 h mortality (CI)	%24 h	ITNs passing *n*/*N* (%)^b^	%KD	%24 h mortality (CI)	%24 h	ITNs passing *n*/*N* (%)^b^	%KD	%24 h mortality (CI)	%24 h	ITNs passing *n*/*N* (%)^b^
	(CI)		Mortality corrected^a^ (CI)		(CI)		Mortality corrected^a^ (CI)		(CI)		Mortality corrected^a^ (CI)	
0	97.1	97.9	97.9	30/30	96.8	99.0	99.0	30/30	98.5	99.2	99.2	30/30
	(94.7–99.6)	(96.0–99.8)	(96.0–99.8)	100%	(94.0–99.6)	(98.2–99.8)	(98.2–99.8)	100%	(95.9–100)	(98.5–99.9)	(98.5–99.9)	100%
6	96.6	89.8	89.8	28/30	97.6	95.3	95.3	29/30	95.3	92.1	92.1	30/30
	(95.1–98.1)	(85.3–94.2)	(85.3–94.2)	93.3%	(96.0–99.3)	(93.3–97.3)	(93.3–97.3)	96.7%	(93.5–97.1)	(89.8–94.4)	(89.8–94.4)	100%
12	98.5	96.6	96.6	30/30	95.0	93.4	93.4	31/31	99.3	98.2	98.2	30/30
	(97.8–99.2)	(95.6–97.6)	(95.6–97.6)	100%	(94.1–95.9)	(92.4–94.4)	(92.4–94.4)	100%	(98.9–99.6)	(97.5–98.9)	(97.5–98.9)	100%
18	97.7	94.6	94.6	28/30	98.8	96.1	96.1	30/30	94.1	68.6	67.6	19/28
	(96.5–99.0)	(92.2–97.2)	(92.2–97.0)	93.3%	(98.2–99.4)	(94.8–97.3)	(94.8–97.3)	100%	(90.1–98.0)	(58.2–79.0)	(56.9–78.2)	67.9%
24	91.4	67.0	65.4	22/30	96.8	95.3	95.2	30/30	82.5	70.6	68.9	10/30
	(86.5–96.4)	(56.0–78.0)	(53.9–76.8)	73.3%	(95.8–97.9)	(93.8–96.8)	(93.7–96.7)	100%	(77.6–87.4)	(64.2–77.1)	(62.3–75.6)	33.3%
30	86.0	50.5	49.5	14/30	94.8	84.0	82.9	25/30	91.3	75.6	72.5	21/29
	(79.8–92.2)	(42.5–58.4)	(41.2–57.7)	48.3%	(91.7–97.8)	(77.3–90.8)	(75.6–90.1)	83.3%	(86.8–95.7)	(66.9–84.4)	(62.7–82.2)	72.4%
36	89.6	45.0	45.0	17/30	96.2	93.5	93.1	49/50	90.5	59.3	58.6	28/50
	(83.9–95.3)	(33.7–56.4)	(33.7–56.4)	56.7%	(95.2–97.2)	(91.8–95.3)	(91.2–95.1)	98.0%	(86.8–94.2)	(50.7–68.0)	(50.0–67.2)	56.0%

^a^Abbott’s correction [24]

^b^Pass based on 24-h mortality criterion only following latest WHO Guidance [30]

### Susceptible strain

Cone bioassays conducted using sensitive strains revealed that Veeralin^®^ ITNs met the WHO efficacy criterion of ≥ 80% 24-h mortality [32] throughout the study period (up to 36 months), MAGNet^®^ up to 18 months, and Olyset^®^ Plus ITNs up to 12 months (Table 4). At 36 months, the 24-h mortality rate of susceptible strains in the cone bioassay was 93.5% (91.8–95.3%) for Veeralin^®^ ITNs, 45.0% (33.7–56.4%) for MAGNet^®^ ITNs, and 58.6% (50.0–67.2%) for Olyset^®^ Plus ITNs.

### Resistant strain

Cone bioassays conducted using resistant strains revealed that Veeralin^®^ ITNs met the WHO efficacy criterion of ≥ 80% 24-h mortality [32] for up to 30 months, whereas Olyset^®^ Plus met the criterion for up to 12 months (Table 5). At 36 months, the 24-h mortality rates of the resistant strains were 68.5% (64.9–72.0%) for Veeralin^®^ ITNs and 58.1% (52.0–64.2%) for Olyset^®^ Plus ITNs.

**Table 5:** Cone bioassays of ITNs at different time points using resistant *Anopheles culicifacies*

Survey (month)	Veeralin^®^ ITNs				Olyset^®^ Plus ITNs			
	%KD	%24 h mortality (CI)	%24 h	ITNs passing^b^ *n*/*N* (%)	%KD	%24 h mortality (CI)	%24 h	ITNs passing^b^ *n*/*N* (%)
	(CI)		Mortality corrected^a^ (CI)		(CI)		Mortality corrected^a^ (CI)	
0	43.9	89.9	89.9	5/5	49.9	90.3	90.3	5/5
	(34.2–53.6)	(87.2–92.6)	(87.2–92.6)	100%	(38.1–61.7)	(89.1–91.6)	(89.1–91.6)	100%
6	85.3	84.5	84.5	23/30	88.2	85.5	85.5	28/30
	(81.5–89.2)	(80.5–88.5)	(80.5–88.5)	76.7%	(86.1–90.3)	(83.8–87.2)	(83.8–87.2)	93.3%
12	93.5	89.2	89.2	30/30	93.6	91	91	30/30
	(92.7–94.2)	(88.2–90.1)	(88.2–90.1)	100%	(92.6–94.7)	(89.9–92.0)	(89.9–92.0)	100%
18	Not done (COVID period)				52.2	71	71	0/10
					(46.7–57.7)	(68.6–73.3)	(68.6–73.3)	0%
24	37.0	61.0	58.3	1/14	38.3	61.8	61.8	1/10
	(27.7–46.4)	(49.0–72.2)	(46.9–69.6)	7.1%	(35.3–41.3)	(52.9–70.8)	(52.9–70.8)	10%
30	69.1	87.0	87.0	10/10	30.1	45.1	45.1	1/12
	(62.5–75.6)	(83.0–90.9)	(83.0–90.9)	100%	(25.1–35.0)	(33.9–56.2)	(33.9–56.2)	8.3%
36	44.7	68.5	68.5	0/10	45.2	58.1	58.1	0/10
	(38.3–51.1)	(64.9–72.0)	(64.9–72.0)	0%	(37.5–52.9)	(52.0–64.2)	(52.0–64.2)	0%

^a^Abbott’s correction [24]

^b^ Pass based on 24-h mortality criterion only following latest WHO guidance [30]

### Adverse events

Adverse events were monitored 1 month after ITN distribution in a subset of households. No serious adverse events were documented, although some users experienced minor side effects, most commonly skin irritation or a burning sensation upon first use (Table 6). Among the 210 households that received 407 Veeralin^®^ ITNs, 87% (490/561) of the individuals reported sleeping under the net the previous night. Among the 198 households that received 188 MAGNet^®^ ITNs, a net usage rate of 78% (319/404 individuals) was reported. Among the 237 households with 411 distributed Olyset^®^ Plus ITNs, 97% net usage was reported (785/808 individuals).

**Table 6:** Adverse events reported by the ITN users after 1 month of use

Symptom	MAGNet^®^ ITNs *n* (%)	Veeralin^®^ ITNs *n* (%)	Olyset^®^ Plus ITNs *n* (%)
Itching of the skin/paresthesia	73/323 (22.6%)	68/495 (13.7%)	20/808 (2.5%)
Facial burning	36/323 (11.1%)	56/495 (11.3%)	12/808 (1.5%)
Sneezing	0/323 (0.0%)	1/495 (0.2%)	2/808 (0.2%)
Runny nose	0/323 (0.0%)	0/495 (0.0%)	2/808 (0.2%)
Headache	1/323 (0.3%)	1/495 (0.2%)	0/808 (0.0%)
Nausea	0/323 (0.0%)	0/495 (0.0%)	1/808 (0.1%)
Eye irritation	43/323 (13.3%)	4/495 (0.8%)	0/808 (0.0%)
Tears from the eyes	0/323 (0.0%)	0/489 (0.0%)	0/808 (0.0%)
Bad smell during use	0/323 (0.0%)	0/489 (0.0%)	7/808 (0.9%)
Any other symptom	0/323 (0.0%)	0/489 (0.0%)	0/808 (0.0%)
Total responses collected	344	561	808

## Discussion

The use of ITNs continues to be one of the most important interventions in malaria prevention [33]. At the community level, data from randomized controlled trials [14, 34, 35] and mathematical models [36, 37] indicate that the effectiveness of ITNs in preventing malaria depends primarily on insecticidal efficacy and secondarily on population coverage. At the individual level, the use of an ITN protects against malaria even in areas where mosquito populations are resistant to the insecticide, due to the physical barrier they provide against bites [38]. The durability (i.e., median functional survival) of ITNs is highly related to physical integrity, because damaged nets are often discarded by users, leading to increased attrition rates [16]. The presence of a net is the most important factor in reducing vectorial capacity [39], and continued bioefficacy maximizes public health benefits. Therefore, the selection of ITNs that are both physically durable and capable of retaining their insecticidal activity for the longest duration is critical for malaria programs [40].

It is well known that the longevity of ITNs is often location-specific [24, 41] and depends on multiple factors, including climate [42], the housing environment, net care behavior [43], net use culture [44], and the physical construction of ITNs [45]. Therefore, malaria control programs should evaluate the durability of different products in their specific settings to choose the most effective and long-lasting option. This will lead to substantial improvements in malaria control [36] and cost savings per person-year of protection [16].

This is the first large-scale durability trial of dual pyrethroid–PBO ITNs conducted under operational field settings in India. This study, which was conducted via a standard protocol and defined guidelines over 3 years, analyzed important outcomes to determine the durability and operational performance of these nets under field conditions by analyzing the median functional survival rate and bioefficacy over time. Moreover, a longitudinal assessment of their performance under field conditions can provide insights to help policymakers choose cost-effective and efficient tools for eliminating malaria nationwide. Additionally, these findings can assist in the selection of the correct ITN replenishment interval to maintain optimal coverage.

The study evaluated Veeralin^®^ ITNs (pyrethroid–PBO net) alongside Olyset^®^ Plus ITNs (pyrethroid–PBO net) and MAGNet^®^ ITNs (pyrethroid-only net). Prior studies have demonstrated that the use of Olyset^®^ Plus ITNs was associated with increased reduction in malaria incidence compared with pyrethroid-only nets in areas where pyrethroid resistance in mosquitoes was conferred by metabolic resistance mechanisms [46, 47]. However, several studies in African countries have reported low durability of < 2 years for PBO nets [30, 48, 49].

This study revealed that Veeralin^®^ ITNs not only achieved a higher functional survival rate than Olyset^®^ Plus ITNs (OR 9.46, *P* < 0.001) did but also demonstrated greater resistance to damage over time, with a generally lower hole surface area. Veeralin^®^ ITNs consistently demonstrated high bioefficacy, as measured by 24-h mortality rates, with most nets passing the bioassay test at all time points. In contrast, Olyset^®^ Plus ITNs and MAGNet^®^ ITNs experienced a more rapid decline in efficacy over time. Compared with Olyset^®^ Plus ITNs, Veeralin^®^ ITNs maintained robust bioefficacy against both pyrethroid-susceptible and pyrethroid-resistant mosquito strains. The inclusion of MAGNet^®^ ITNs as a pyrethroid-only control highlighted the enhanced performance of both pyrethroid–PBO nets against resistant mosquito strains in this setting. Veeralin^®^ ITNs, in particular, were able to sustain optimal insecticidal activity for up to 30 months, whereas Olyset^®^ Plus ITNs maintained this level for up to 12 months.

In addition to their high physical durability, the sustained bioefficacy of pyrethroid–PBO ITNs has significant importance in the face of emerging pyrethroid resistance in malaria vectors. This study highlighted that Veeralin^®^ ITNs have effective insecticidal activity against resistant strains for a prolonged period. Such performance is critical for malaria control programs, especially in areas where emerging pyrethroid resistance undermines the effectiveness of standard pyrethroid ITNs [12]. The cost of intervention and replacement after the prescribed life of use is an additional consideration for large-scale public health interventions. Studies from Tanzania [16] and Nigeria [41] suggest that improved net longevity not only enhances malaria prevention outcomes but also reduces the frequency of net replacement, thereby optimizing resource allocation, which is crucial for policy implementation, especially in the current climate of limited donor funding [50]. The ITNs in the study were identified as having relative longevity that could, in the long run, translate into significant economic advantages by reducing replacement costs and ensuring sustained community protection over time.

Many factors, including residing in low malaria transmission areas, living in rural settings, having a large family, engaging in indoor cooking practices, and having lower socioeconomic status, are associated with shorter functional survival times of ITNs [51] and were observed in the study area (Supplementary Table 3). Despite these challenges, however, most ITNs maintain bioefficacy through the 2 year of use [52]. According to the WHO criteria, long-lasting insecticidal nets are those that remain in use under serviceable conditions and are efficacious for 3 years [53]. In this study, Veeralin^®^ ITNs remained in use and serviceable for more than 3 years and were also found to retain insecticidal effectiveness against susceptible mosquitoes for 3 years and resistant mosquitoes for up to 30 months.

Despite careful planning and implementation, the study faced a few limitations. The COVID-19 pandemic caused significant disruptions in field operations, and post-pandemic population migration hindered follow-up and timely retrieval of ITNs for assessing bioefficacy and durability for 1–2 years. Additionally, the unavailability of residents and extreme climatic conditions at the study site led to delays in extracting ITNs from the field. However, efforts were made to approach the same household at least twice to retrieve the ITNs.

## Conclusions

Overall, this study contributes to the growing body of knowledge supporting the use of dual pyrethroid–PBO-based ITNs as a critical component of integrated malaria control strategies in areas of low to moderate pyrethroid resistance. In the Indian geographic context, Veeralin^®^ fulfilled the WHO criteria for durability, maintaining serviceable physical integrity in 96.1% of nets over 3 years, so they can be categorized as long-lasting insecticidal nets. The nets retained optimal insecticidal efficacy against both pyrethroid-resistant and pyrethroid-susceptible *Anopheles* mosquito populations throughout the evaluation period, supporting their potential role in advancing malaria elimination targets slated to be achieved by the end of the decade.

## Supplementary Information

Supplementary Material 1.

## Data Availability

Data supporting the main conclusions of this study are included in the manuscript.

## Abbreviations

ITNs: Insecticide-treated nets
PBO: Piperonyl butoxide
NFME: National Framework for Malaria Elimination
PQ: Pre-qualified
THSA: Total hole surface area
WHO: World Health Organization
KD: Knockdown
SD: Standard deviation
IQR: Interquartile range
CI: Confidence interval
GOI: Government of India
