# Supplementary material for: Durability study of Veeralin®, Olyset® Plus and MAGNet® insecticide-treated nets (ITNs) in Kurnool district, Andhra Pradesh, India
Source: Parasit Vectors. 2025 Oct 29;18:441. doi: 10.1186/s13071-025-07016-2 (PMC12574002; doi:10.1186/s13071-025-07016-2)
Supplement: Supplementary file 1 — Supplementary Material 1. [file 13071_2025_7016_MOESM1_ESM.docx]

**Supplementary Tables**

**Supplementary Table 1**: Study Objectives and outcome measurements over the three year study

| **Test** | **Objective** | **Outcome measure(s)** |
| --- | --- | --- |
| **QUESTIONNAIRE** | Record the user conditions and adverse events | Proportion of participants in each category |
| **ATTRITION** | Evaluate the functional net loss of ITN | Net survivorship (attrition rate) |
| **ITN INTEGRITY** | Evaluate functional survival (fabric integrity) of ITN | Holed surface area, proportion serviceable |
| **BIOASSAYS** | Determine the biological efficacy of ITN | % 24 hour Mortality (95% CI) in cone test |

**Supplementary Table 2**: Resistance profile of the laboratory reared mosquitoes (% 24 hour mortality)

| **24-hour mortality** | Alpha-cypermethrin | | | | | Permethrin | | | |
| --- | --- | --- | --- | --- | --- | --- | --- | --- | --- |
|  | 2019 | 2020 | 2021 | 2022 | 2024 | 2020 | 2021 | 2022 | 2024 |
| **Susceptible** | 100% | 100% | 100% | 100% | - | - | 100% | 100% | _ |
| **Resistant** | 60.85% | 67.2% | - | 84.85% | 98% | - | - | - | 91% |

**Supplementary Table 3:** Demographic details of the study households, Kurnool, India

| Factor | Veeralin® ITN | Olyset® Plus ITN | MAGNet® ITN |
| --- | --- | --- | --- |
| Number of participants | 5958 | 4385 | 5602 |
| Number of households | 1457 | 1282 | 1447 |
| **Household size: Mean ± SD** | 3.73 ± 1.571 | 3.32 ± 1.456 | 3.60 ± 1.608 |
| **Gender of the head of the household** | | |  |
| Male | 1188 | 1023 | 1220 |
| Female | 267 | 258 | 226 |
| Transgender | 2 | 1 | 1 |
| **Age of head of the household: Mean ± SD (n)** | 42.66 ± 14.883 | 42.55 ± 12.546 | 43.87 ± 14.545 |
| **Age distribution of family members** | | |  |
| <5 years | 356 | 320 | 354 |
| 5 – 14 years | 1060 | 820 | 1088 |
| >= 15 years | 4542 | 3245 | 4160 |
| **Highest education of head of the household** | | |  |
| Not able to read and write | 820 | 974 | 937 |
| Primary | 331 | 100 | 251 |
| Secondary | 227 | 150 | 182 |
| Tertiary | 66 | 30 | 61 |
| Others | 13 | 28 | 16 |
| **Material of the roof** | | |  |
| Grass | 142 | 183 | 115 |
| Thatch | 961 | 961 | 1074 |
| Cement / concrete | 198 | 90 | 123 |
| Iron sheets | 12 | 2 | 7 |
| Others (Mud slabs) | 144 | 95 | 128 |
| **Material of the walls** | | |  |
| Burnt bricks | 1154 | 1094 | 1219 |
| Cement bricks | 83 | 20 | 71 |
| Mud | 87 | 20 | 33 |
| Sticks / Grass | 67 | 79 | 67 |
| Others | 66 | 69 | 57 |
| **Material of the Floor** | | |  |
| Cement | 10 | 10 | 2 |
| Earth or sand | 24 | 24 | 23 |
| Tiles | 335 | 216 | 357 |
| Others – Wood, Plastic Sheets, Asbestos Sheet, Coconut and Palm Leaf etc. | 1088 | 1032 | 1065 |
| Total Net Distributed | 2664 | 2246 | 2660 |
